# Vitamin D supplementation for patients with multiple sclerosis treated with interferon-beta: a randomized controlled trial assessing the effect on flu-like symptoms and immunomodulatory properties

**DOI:** 10.1186/1471-2377-13-60

**Published:** 2013-06-14

**Authors:** Daniel Golan, Basheer Halhal, Lea Glass-Marmor, Elsebeth Staun-Ram, Orit Rozenberg, Idit Lavi, Sara Dishon, Mira Barak, Sophia Ish-Shalom, Ariel Miller

**Affiliations:** 1 Division of Neuroimmunology & Multiple Sclerosis Center, Carmel Medical Center, 7 Michal Street, Haifa, 34362, Israel; 2 Department of Neurology, Carmel Medical Center, Haifa, Israel; 3 Central Laboratory of Haifa and Western Galilee, Clalit Health Services, Haifa, Israel; 4 Department of Community Medicine & Epidemiology, Carmel Medical Center, Haifa, Israel; 5 Rappaport Faculty of Medicine, Technion-Israel Institute of Technology, Haifa, Israel

**Keywords:** Flu-like symptoms, Interferon-β, IL-17, Multiple sclerosis, PTH, Quality of life, Safety, Vitamin D

## Abstract

**Background:**

Flu-like symptoms (FLS) are common side effects of interferon beta (IFN-β) treatment in patients with Multiple Sclerosis (PwMS) and are associated with post-injection cytokine surge. We hypothesized that vitamin D3 supplementation would ameliorate FLS by decreasing related serum cytokines’ levels.

**Methods:**

In a randomized, double blind study of 45 IFNβ-treated PwMS, 21 patients were assigned to 800 IU of vitamin D3 per day (low dose), while 24 patients received 4,370 IU per day (high dose) for one year. FLS were assessed monthly by telephonic interviews. Serum levels of 25-hydroxy-D (25-OH-D), calcium, PTH, IL-17, IL-10 and IFN-γ were measured periodically. EDSS, relapses, adverse events and quality of life (QoL) were documented.

**Results:**

25-OH-D levels increased to a significantly higher levels and PTH levels decreased in the high dose group. There was no significant change in FLS. IL-17 levels were significantly increased in the low dose group, while patients receiving high dose vitamin D had a heterogeneous IL-17 response. No significant differences in relapse rate, EDSS, QoL, serum IL-10 and IFNγ were found. Hypercalcemia or other potential major adverse events were not observed.

**Conclusion:**

Vitamin D supplementation to IFN−β treated PwMS, at the doses used, seems safe and associated with dose-dependent changes in IL-17 serum levels, while not affecting IFN−β related FLS.

**Trial registration:**

ClinicalTrials.gov ID: NCT01005095

## Background

Flu-like symptoms (FLS) are well known side-effects of interferon beta (IFN-β) treatment for patients with Multiple Sclerosis (PwMS). Over half of the treated patients experience systemic adverse-effects after treatment initiation [1]. Although these symptoms tend to diminish with time, some patients experience ongoing FLS and are unable to tolerate the drug. FLS are often the reason for inadequate adherence to medication and for treatment discontinuation, which may reach beyond 20% of those who stopped using IFN-β due to adverse effects [2].

FLS have been shown to be related to a post IFN-β injection surge of cytokines, such as IL-6, TRAIL and IP-10/CXCL2 by peripheral blood mononuclear cells [3,4]. The accumulating evidences that vitamin D may down regulate the secretion of these cytokines [5-7], led us to hypothesize that vitamin D supplementation may ameliorate IFN-β-induced FLS.

The usefulness of combining vitamin D and IFN-β for MS treatment is yet to be determined. Recently, an observational study of MS patients suggested that serum 25-hydroxy-vitamin D (25-OH-D) level was associated with a reduced relapse risk only among patients treated with IFN-β [8], while in another patient cohort no association between 25-OH-D and disease activity was detected among IFN-β -treated MS patients [9]. We have lately reported that high dose vitamin D supplementation is associated with decreased melatonin secretion in IFN-β treated PwMS [10], implying that melatonin may be one of the mediators of 25-OH-D action on the immune system. Levels of melatonin as well as of other mediators may potentially explain some of the variation in the clinical and immunological response to vitamin D.

Furthermore, a clinical trial of vitamin D3 as an add-on to IFN-β treatment in MS patients demonstrated a reduction in MRI disease activity compared to a placebo group [11]. However this effect has yet to be replicated in larger cohorts.

The primary objective of the present study was to test whether vitamin D supplementation may ameliorate IFN-β-induced FLS. Secondary objectives were to evaluate the safety and tolerability of vitamin D in two different regimens, to determine the extent it influences serum 25-OH-D and PTH levels and to assess the effect of vitamin D supplementation on IFN-β- treatment efficacy, determined by relapse rate and EDSS, as well as on the serum levels of cytokines associated with immune-mediated diseases such: IL17, IFNγ and IL-10, proposed to be associated with MS disease fluctuating activity [12,13].

## Methods

### Patients

Patients with clinically and laboratory definite Relapsing Remitting MS according to the revised McDonald’s criteria [14], who attended our clinic at the MS Center at Carmel Medical Center, Haifa for routine follow-up from November 2010 to March 2011 were offered to participate (n = 49). The study was approved by the local Helsinki Ethics Committee of Carmel Medical Center, and all participants gave their written informed consent. The trial was registered at ClinicalTrials.gov ID: NCT01005095.

Inclusion Criteria were: Age ≥ 18 years ; assigned to initiate IFN-β treatment (n = 1) or patients who continued to suffer from FLS beyond 4 months of treatment with IFN-β (n = 44); 25-OH-D blood levels < 75 nmol/l and EDSS score < 7. Exclusion Criteria consisted of abnormalities of vitamin D related hormonal system other than low dietary intake or decreased sun exposure (such as: intestinal malabsorption, chirosis, nephrotic syndrome, hyperthyroidism, creatinine clearance of less than 40 ml/min, rickets, hypoparathyroidism, hypercalcemia at baseline, known malignancy, Granulomatous disorders and lymphomas). We also excluded patients who took medications that influence vitamin D metabolism, namely, Orlistat, anticonvulsants, Rifampin, Isoniazide, Ketoconazole, 5FU and Leucovorin. Patients with conditions of increased susceptibility to hypercalcemia were excluded too: known arrhythmia, heart disease, nephrolithiasis and treatment with Digitalis or Hydrochlorothiazide. Pregnant women were not included.

Participation after enrolment was terminated in case of withdrawal of IFN-β treatment for any reason, pregnancy, hypercalcemia or patient’s decision.

### Study groups, randomization and supplementation regimens

The study included two interventional groups: a “high dose” group assigned to oral treatment of one bottle containing 75,000 IU of vitamin D3 solution every 3 weeks in addition to 800 IU of vitamin D3 by daily tablets (total of 4370 IU/d); and a “low dose” group receiving one bottle of placebo solution every 3 weeks besides 800 IU of vitamin D3 by daily tablets (total of 800 IU/d). The vitamin D content in solution bottles and tablets was quantitatively evaluated by the manufacturers and the results were within the specification. The assignment to groups was randomly set in advance, according to recruitment order. Vitamin D supplementation was double-blind in this study – both participants, treating physician and investigators were unaware of the ingredients of the solution bottles.

After recruitment, patients were required to adhere to study vitamin D products and to refrain from taking any other vitamin D formulations. The half-life of 25-OH-D is about 2–3 weeks [15]. Basic pharmacology suggests that after a period of 5 half-lives, serum levels are at a new steady state [16]. Therefore, by 3 months from enrolment, serum 25-OH-D levels represent the new steady state equilibrium, and are not influenced by pre study vitamin D intake.

### Clinical follow-up

FLS were assessed by phone interviews at least once per month, and if participants were available, twice monthly. Patients were asked to refer to their last IFN injection and to rate the extent of FLS on a Likert scale. A score from 0 (not at all) to 5 (very strong), was given to each FLS component: myalgia, chills, headache, malaise and fatigue, exacerbation of neurologic symptoms, sweating and difficulty to function compared to the time before injection. The scores of individual components were summed, yielding a total FLS score ranging from 0 to 35 [3,4]. If a patient was interviewed twice at a given month, the higher score was used for analysis, in order to be sure that worst possible symptoms are recorded and to diminish the influence of transient fluctuations. Patients were allowed to use pain relieving medications during the study. Information about the use of these medications over 6 months was obtained in the interviews.

Complete neurologic examination and EDSS scoring were done at baseline, after 6 months and at 1 year. Relapses were recorded throughout the study. When new MS symptoms were reported in the phone interview, the patient was invited to further evaluation at the clinic. Relapses were defined as new neurologic deficits, lasting longer than 24 hours, with no evidence of an infection [14]. All relapses were confirmed by objective neurologic examination.

Health related quality of life (HRQoL) was evaluated at baseline and at the completion of the study, using the ‘Functional assessment of MS’ questionnaire (FAMS), which is divided to six subscales: mobility, symptoms, emotional well-being (depression), general contentment, thinking/fatigue, and family/social well-being [17]. The total FAMS score is calculated by summing all subscale scores, ranging from 0 to 232. High score denotes decreased QoL.

At each clinic visit during the trial patients were asked to report any new adverse event that might have been somehow related to vitamin supplementation.

### Laboratory evaluation

Blood samples were collected at baseline and after 3, 6, and 12 month of vitamin D supplementation, processed for serum within 45 minutes from its withdrawal and stored at −80°C until analysis. Serum 25-OH-D and calcium levels were measured at baseline, 3, 6 and 12 month, while serum PTH measured at baseline and 3 months.

Vitamin D total assay uses chemiluminescent immunoassay (CLIA) technology and was tested on the Liaison analyzer (DiaSorin S.p.A., Italy) for the quantitative determination of 25-hydroxy-vitamin D.

PTH assay uses chemiluminescent immunoassay (CLIA) technology and was tested on the Immulite 2000 analyzer (Siemens AG) for intact Parathyroid hormone quantitative measurement.

Serum cytokine levels (IL10, IFNγ and IL17) were measured at baseline and after 3 months. Commercial ELISA kits were used: IL-10 (Quantikine HS, R&D systems, USA), IFNγ (eBioscience, USA) and IL-17 (Enzo Life sciences, USA). Cytokine concentrations were determined as described by the manufacturers’ user manual. All measurements from each patient were performed in the same plate in the same assay. Intra-assay coefficient of variations (CV) was ≤ 10% for each plate.

### Statistical analysis

Data analysis was performed using the SPSS statistical package (SPSS Inc., Chicago, IL, ‘USA). Comparisons of patients’ characteristics between study groups at baseline were done by Student’s t-test or Mann–Whitney test, according to data distribution. Changes in 25-OH-D levels, calcium and EDSS were analyzed by ANOVA with repeated measures. PTH and IL-17 levels as well as FAMS scores at baseline and after 3 months were compared by paired Student’s t-test. Alterations in IFN-γ levels were analyzed by Wilcoxon Signed Rank test.

Comparisons of FLS scores at various times were done by mixed model ANOVA. This procedure takes into account the intra-correlation of repeated measurements carried out at the same subject and does not exclude subjects with incomplete data at follow-up. FLS scores according to the use of pain relieving medications were compared by Mann–Whitney test. Comparisons between proportions of participants who had a relapse were done by Chi square test. Summary statistics are reported as mean ± Standard deviation and as median (range).

Annualized relapse rates were calculated as number of relapses divided by the sum of person-years under follow-up. 95% Confidence intervals were calculated using normal approximation. Relapse rates were compared by Mid-P exact test, using the Open Epi epidemiologic calculator (Atlanta, GA, USA).

According to our initial power analysis, a sample of 100 patients, 50 in each vitamin D dosage group, was needed to provide 80% power to detect a mean change in the FLS score of 1 point, using t-tests for unpaired values, alpha 0.05, standard deviation 1.5 points. After 45% intended enrollment and 1 year of follow up, an interim analysis of the data was conducted and the probability of finding a significant difference in FLS scores at study end, assuming the trend to date would continue, was calculated with the intent to close enrollment, if the probability decreased below 50%.

## Results

**Figure 1:**
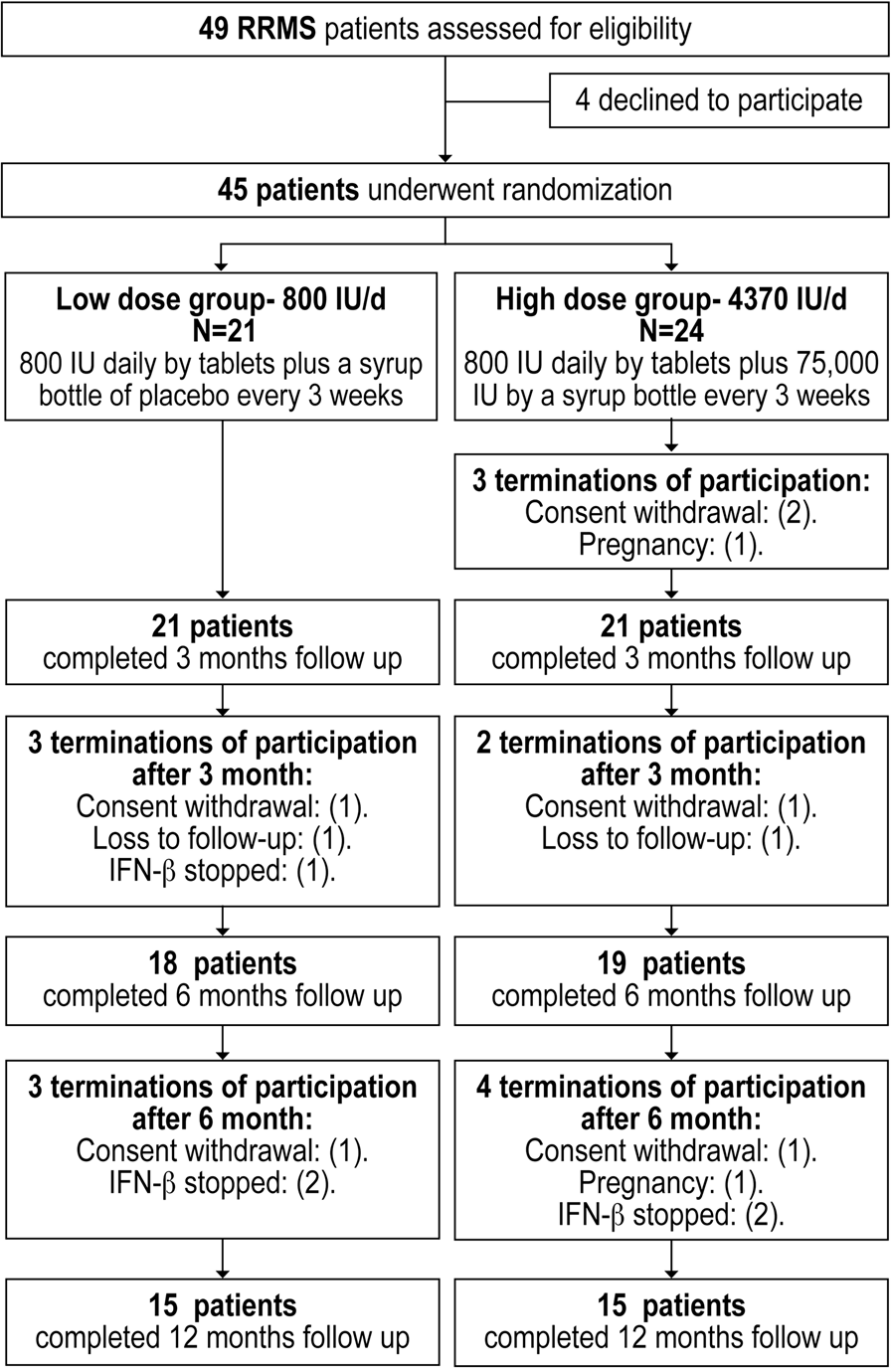
Study flow chart.

Flow chart of the study is shown in Figure 1. Patients’ characteristics at recruitment are provided in Table 1. All participants, except one patient, were lengthy treated with IFN-β, and were experiencing FLS despite continuous use of IFN-β for more than 4 months.

**Table 1:** **Patients’ characteristics at baseline **^**a**^

	***Low dose group***	***High dose group***	***p value***
	***800 IU/d***	***4,370 IU/d***	
	***N = 21***	***N = 24***	
***Age (years)***	44.7 ± 10.7	43.1 ± 12.3	0.7 ^b^
	43.6 (26.7-63.9)	43.1 (21.7-63.7)	
***Gender (Male/Female)***	F 13 (62%)	F 19 (79%)	0.2 ^c^
	M 8 (38%)	M 5 (21%)	
***Time from MS diagnosis (years)***	9.3 ± 7.4	6 ± 5.7	0.08 ^d^
	8.1 (0.4-32.9)	3.4 (0.3-19.1)	
***IFN-β treatment duration (months)***	61.3 ± 46	38.9 ± 32.5	0.1 ^d^
	59.1 (3.1-144.1)	23.4 (1.2-96.7)	
***EDSS***	3.6 ± 2.2	2.9 ± 2.0	0.4 ^d^
	4.5 (0–7)	2.5 (0–7)	
***Patients with relapses during the year preceding randomization***	6/21 (28.6%)	8/24 (33.3%)	0.7 ^c^
***Annualized relapse rate during the year preceding randomization***	0.38 ± 0.26	0.28 ± 0.23	0.6 ^e^

a. Summary statistics are mean ± standard deviation and median (range).

b. Student’s t-test.

c. Chi square test.

d. Mann–Whitney test.

e. Mid-P exact test.

### Serum vitamin D, PTH and calcium levels

**Figure 2:**
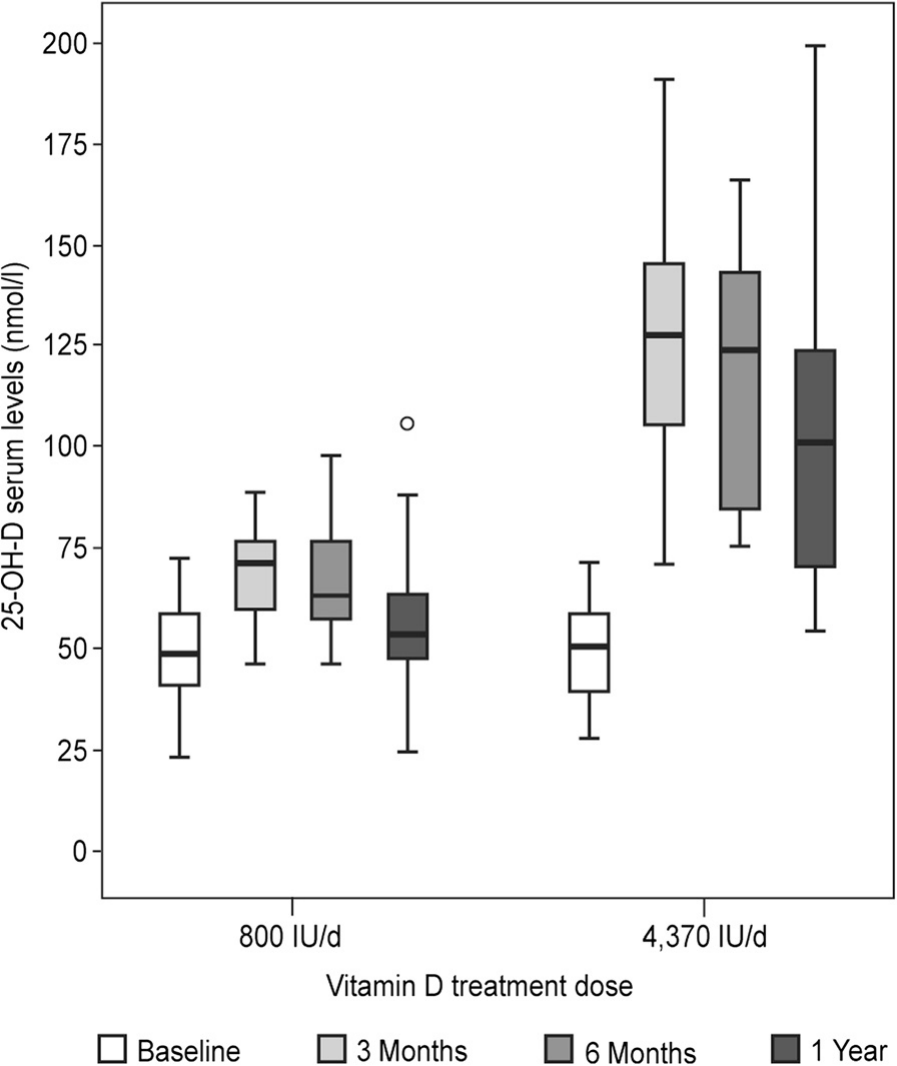
**25-OH- vitamin D serum levels**. Serum 25-OH-D (25-hydroxy-vitamin D) was measured at baseline, 3, 6 and 12 month using a chemiluminescent immunoassay. The high dose supplementation resulted in significantly higher serum 25-OH-D levels compared to low dose throughout the follow up (P < 0.001). With high dose, 25-OH-D levels were significantly above baseline at all time points (P ≤ 0.01), while low dose resulted in significantly increased 25-OH-D levels compared to baseline only at 3 months (P = 0.006) and at 6 months (P = 0.04).

Serum 25-OH-D levels at baseline, 3 months, 6 months and 1 year in the two dosage regimens are presented in Figure 2. After 3 months of supplementation, 25-OH-D levels significantly increased from 48 ± 14.2 to 68 ± 11 nmol/l and from 48.2 ± 13.9 to 122.6 ± 32 nmol/l in the low and high dose groups, respectively. The high dose supplementation resulted in significantly higher serum 25-OH-D levels compared to low dose throughout the follow up (P < 0.001). While in the high dose group, 25-OH-D levels were significantly above baseline at all time points (P ≤ 0.01), in the low dose group a significant increase was found only after 3 months (P = 0.006) and 6 months (P = 0.04), whereas 25-OH-D levels were not significantly different from baseline by the end of 12 month of follow up.

PTH decreased in the high dose group, while it did not significantly change with low dose vitamin D (Table 2). Serum calcium levels remained stable and within normal range in both dosage groups, throughout the study (Table 2).

**Table 2:** **Clinical and Laboratory results **^**a**^

***Study group***	***Low dose group***					***High dose group***				
	***800 IU/day***					***4370 IU/day***				
***Parameter***	***Baseline***	***3Months***	***6 Months***	***End***	***p-value***	***Baseline***	***3Months***	***6 Months***	***End***	***p-value***
***Annual Relapse rate***	0.38±0.26	--	--	0.34±0.27	0.88 ^b^	0.28±0.23	--	--	0.51±0.34	0.32 ^b^
***EDSS***	3.6±2.2	--	3.6±2.1	3.6±2.3	0.1^c^	2.9±2.0	--	3.4±2.3	3.3±2.4	0.26 ^c^
	4.5		2.8	3.0		2.5		3	2.5	
	(0-7)		(0-7)	(0-7)		(0-7)		(0-7)	(0-7)	
***Quality of life score (FAMS)***	145.6±30.8	--	--	142.7±32.5	0.68 ^d^	147.9±38.6	--	--	146.6±45.5	0.84 ^d^
	138			134		158			150	
	(103-205)			(94-212)		(85-211)			(71-225)	
***Serum calcium (mg/dl)***	9.5±0.4	9.7±0.3	9.5±0.4	9.5±0.5	0.4 ^c^	9.6±0.3	9.7±0.2	9.6±0.2	9.4±0.5	0.2 ^c^
	9.5	9.6	9.5	9.4		9.5	9.5	9.6	9.4	
	(8.7-10.2)	(9.2-10.2)	(9-10.3)	(8.9-10.3)		(9.2-10.1)	(9.4-10.1)	(9.2-10.1)	(8.6-10.1)	
***PTH (pg/ml)***	36.7 ±17.1	31.6±11.5	--	--	0.17 ^d^	30.4±13.5	25.0±10.0	--	--	0.04 ^d^
	35.5	29.4				31.1	21.7			
	(11.5-77.4)	(12.8-53.3)				(8.3-49.9)	(6.4-41.4)			
***IFN-γ (pg/ml)***	0.20±0.22	0.14±0.2	--	--	0.13 ^d^	0.51±1.1	0.58±1.3	--	--	0.7 ^d^
	0.08	0.03				0.05	0.07			
	(0-0.61)	(0-0.77)				(0-4.4)	(0-5.8)			
***IL17 (pg/ml)***	4.01±3.99	9.14±9.9	--	--	0.04 ^d^	5.83±6.1	6.38±6.7	--	--	0.75 ^d^
	3.74	7.8				4.3	3.4			
	(0-11)	(0-38.3)				(0-19)	(0-20.8)			
***IL10 (pg/ml)***	undetected	undetected	--	--	--	undetected	undetected	--	--	--

a. Summary statistics are mean ± standard deviation and median (range).

b. Mid-P exact test.

c. ANOVA with repeated measures*.*

d. Paired Student’s t-test.

### Flu-like symptoms

**Figure 3:**
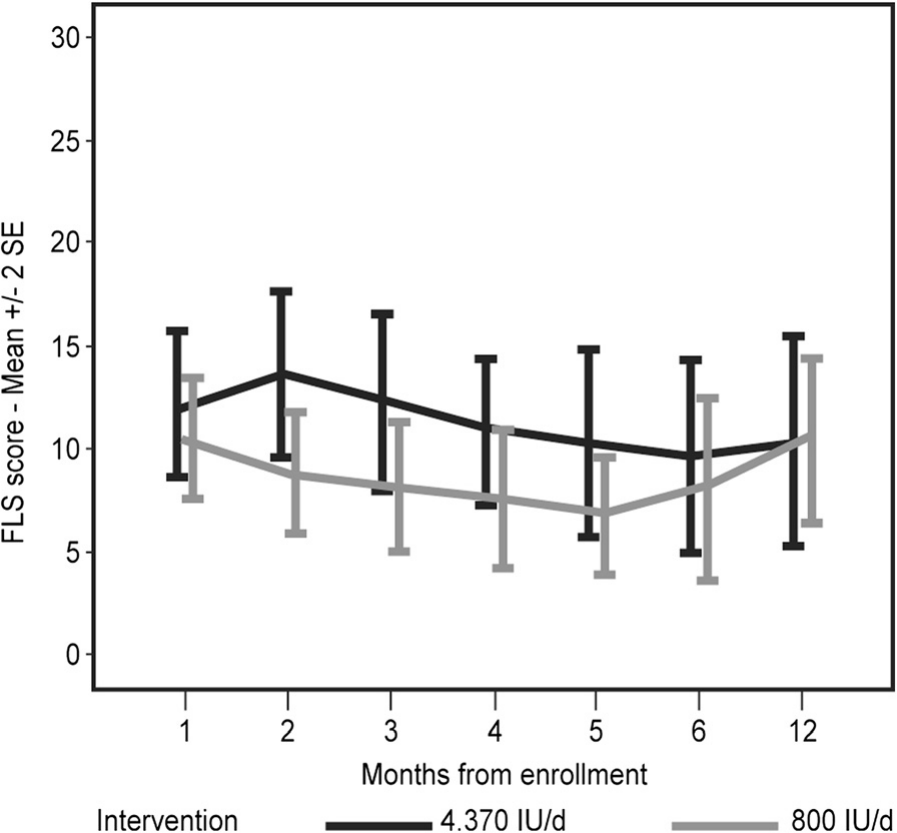
**Flu like symptoms score by time from enrollment to vitamin D supplementation.** FLS were assessed by monthly phone interviews. Patients rated the extent of FLS on a Likert scale (a total FLS score ranging from 0 to 35). No significant change in FLS severity was noted in both dosage groups.

FLS scores of the patients are shown in Figure 3. No significant change in FLS severity was noted. Comparisons of FLS scores between patients who received high and low doses of vitamin D indicated insignificant differences at all time points.

**Figure 4:**
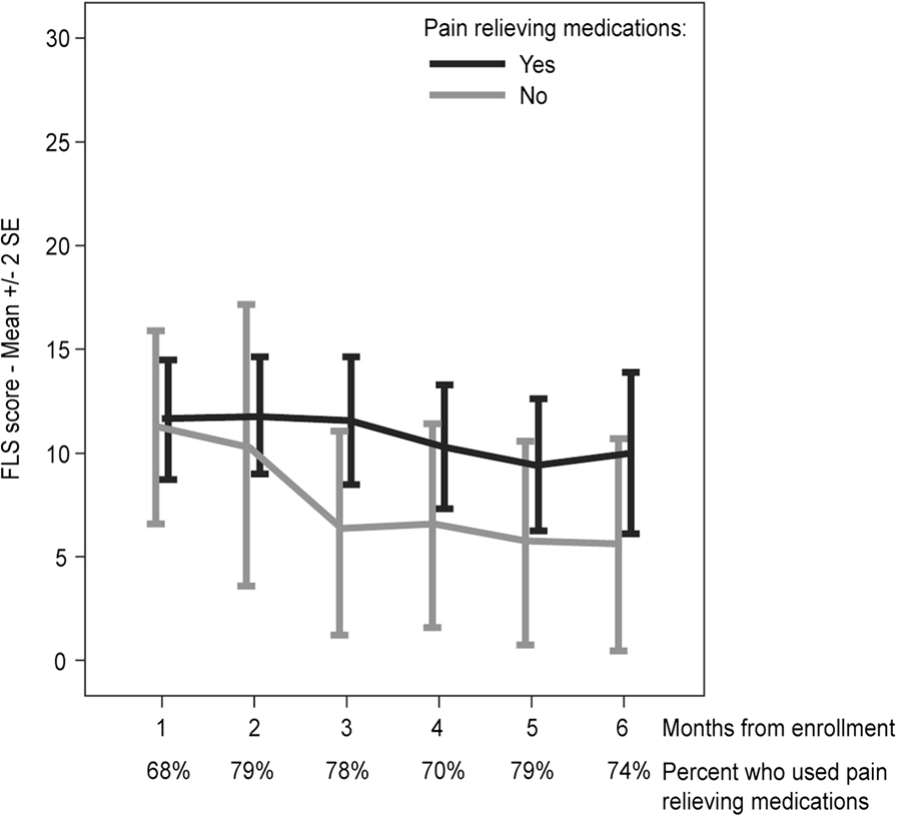
**Flu like symptoms score and use of pain relieving medications**. No statistically significant differences were found in FLS scores between patients who used analgesics and patients who did not use analgesics, regardless of vitamin D dose.

The majority of participants (68%-79% depending on month) opted to use pain relieving medications, namely Ibuprofen, Paracetamol or Dipyrone. As could be expected, mean FLS scores were higher in participants who needed analgesics, but the differences did not reach statistical significance, regardless of vitamin D dose (Figure 4). Notably, most patients were using analgesics on a regular and frequent basis, therefore FLS data without pain relieving medications came from different patients at different time points.

An interim data analysis was conducted and the data were reviewed for futility. At 45% enrollment, the conditional power of detecting a difference in FLS given the current trend was calculated to be below 5%. That is to say, there was less than 5% probability to find a significant difference in FLS between the groups, if the study would continue to the planned number of recruitments. Consequently, the trial was closed to further enrollment.

### MS clinical parameters

Clinical parameters are presented in Table 2. No significant changes in FAMS score, EDSS or relapse rates were noted in either group. The statistically insignificant increased relapse rate in the high dose group stemmed from one patient who had 3 relapses after enrolment compared to none at the year prior randomization.

### Serum cytokines

**Figure 5:**
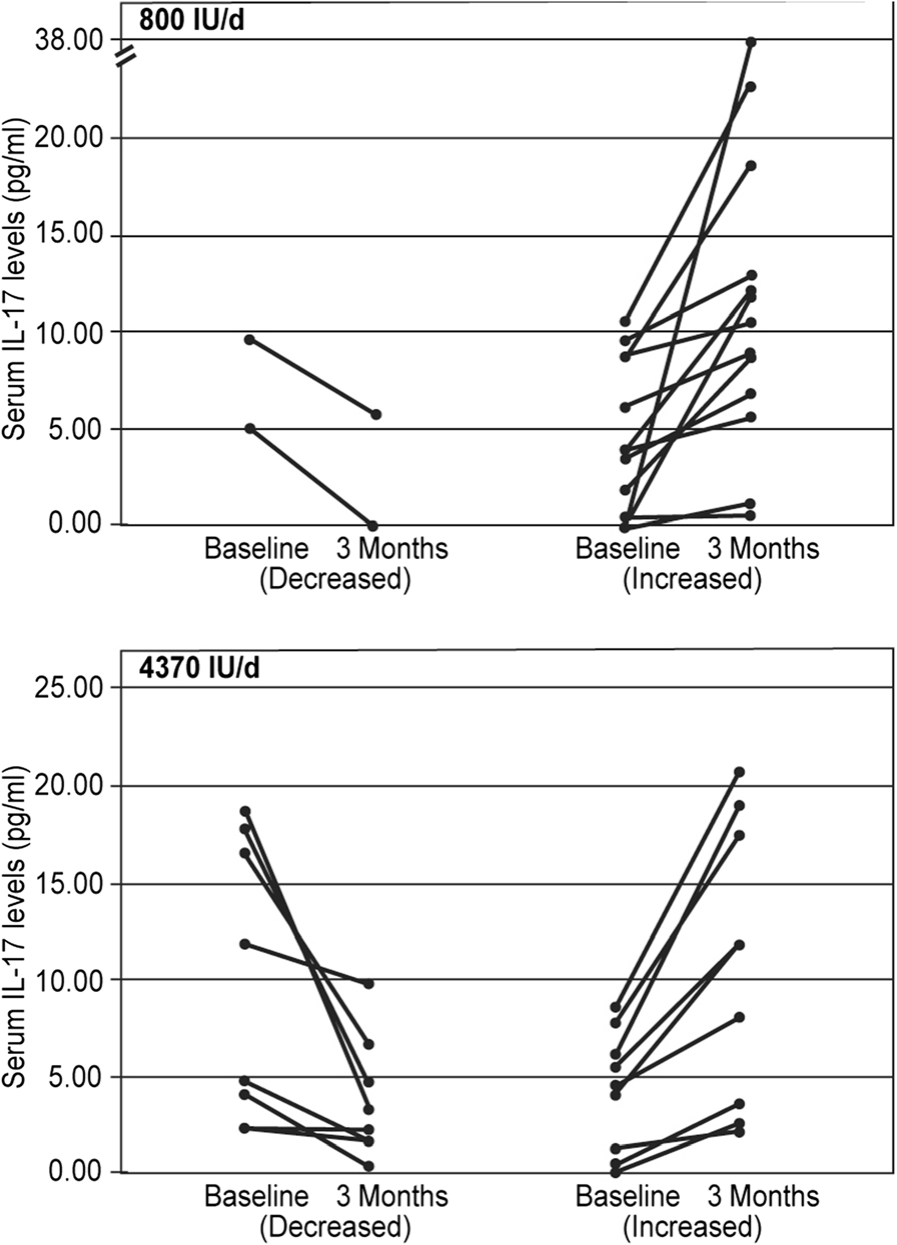
**Changes in IL-17 serum levels after 3 months supplementation with vitamin D.** A significant increase in serum IL-17 was detected at 3 month in the low dose vitamin D group (p = 0.037). Serum IL-17 response to high dose vitamin D was heterogeneous. 3 patients in each dosage group had IL-17 levels below the detection threshold of the ELISA kit at both time points (not shown in the figure).

Cytokine levels are shown in Table 2. Serum IL-17 measurements were available at baseline and after 3 months of vitamin D supplementation for 18 patients in the low dose group and for 20 patients in the high dose group (Figure 5). 3 patients in each dosage group had IL-17 levels below the detection threshold of the ELISA kit at both time points (these entries are not shown in Figure 5, but were included as zero for data averaging in Table 2). A significant increase from 4.01 ± 3.99 to 9.14 ± 9.9 pg/ml in serum IL-17 was detected at 3 month in the low dose vitamin D group (p = 0.037). Serum IL-17 response to high dose vitamin D was non homogenous. While 8 patients (40%) had decreased serum IL-17 levels after 3 months, 9 patients (45%) had increased IL-17 levels after the same period of high dose supplementation. The other 3 patients (15%) had IL-17 levels below the detection threshold at both time points. We are not aware of any convincing scientific data that indicate which magnitude of serum IL-17 change is deemed clinically meaningful. Therefore, an increase in IL-17 serum levels was defined as a positive change, while a decrease was defined as negative change, regardless of magnitude (Figure 5).

Non significant changes were found in IL-10 and IFN-γ levels (Table 2).

### Adverse events

There was no occurrence of hypercalcemia and no reports on new adverse event which could be vitamin D supplement related. None of the patients stopped participation due to adverse events.

## Discussion

The role of vitamin D in MS pathogenesis is currently at the focus of multidisciplinary research initiatives. Prior research evaluated the role of vitamin D deficiency as a risk factor for development of MS and as a modifier of its clinical course [18] as well as of common symptoms of PwMS, such as pain and depression [19,20]. The interaction between IFN-β and vitamin D in terms of their combined efficacy was also previously studied both clinically [8,9,11] and in an animal model [21], with yet conflicting results.

In the current work we focused on a different aspect of the vitamin D – IFN-β interaction, namely: the effect on FLS, a bothering, sometimes debilitating, adverse-effect of IFN-β treatment. We hypothesized that vitamin D may ameliorate these symptoms, by modulating a post-injection cytokine surge. In the present randomized, double-blind study, vitamin D supplementation did not affect IFN-β-induced FLS. Futility analysis showed that this conclusion was unlikely to change with recruitment of more patients.

The desired 25-OH-D level for the general population is derived from epidemiologic studies of the relationship between this level and the risk for osteoporosis. The controversy in translating the data to practice is reflected in conflicting recommendations of current guidelines. While the American institution of medicine recommends a target of 50 nmol/l [22], the American society of endocrinology defines levels below 75 nmol/l as insufficient and below 50 nmol/l as deficient [23]. Additionally, since some findings suggest that high dose of vitamin D might have a direct toxic effect [24], the optimal level range for vitamin D has not yet reached consensus.

Our data show that the high dosage regimen is more effective in achieving 25-OH-D serum level above 75 nmol/l in MS patients. After 6 months, only 25% of the low dose vitamin D group reached serum levels of 75nmo/l or higher, compared to 100% of the high dose group. By the end of the study only 13% of those treated with 800 IU/d compared to 60% of those treated with 4,370 IU/d reached the 75 nmo/l target.

Our patients’ average EDSS score was about 3.5, implying that most of them were not housebound and could have been influenced by outdoor sun exposure. Therefore, the observed decline in 25-OH-D from 6 months to 12 months in both groups can be partly attributed to winter conditions at the 12 month visit. Annual variation in 25-OH-D levels, with decreased levels at winter, has previously been reported in Israel [25]. All patients confirmed their adherence to the study regimen in every phone conversation as well as in each clinic visit. However, attrition in compliance can’t be ruled out and might have contributed to the decreased 25-OH-D levels after 12 months in both dosage groups.

As of today, only few, small scale randomized controlled trials of vitamin D supplementation have been completed, and with conflicting results. Burton et al. [26] randomized 49 MS patient to no supplementation or escalating vitamin D doses up to 40,000 IU/day over 28 weeks followed by 10,000 IU/day for 12 weeks, thence down-titrated to 0 IU/d until the end of 52 weeks of follow up. Treatment group patients appeared to have fewer relapses and a persistent reduction in T-cell proliferation compared to controls. In another study, 23 patients were randomized to 6000 IU/d vitamin D or placebo. After 6 months follow up no significant differences were detected in various MRI endpoints, and there were more relapses in the high dose group [27].

Recently, Soilu-Hanninen et al. [11] reported a 1 year, double blind, placebo controlled, randomized study in 66 MS patients with vitamin D3 as an add-on treatment to IFN-β. Patients in the vitamin D group showed improved MRI outcomes, with no significant differences in clinical outcomes. Similarly, a 96-week randomized controlled trial of 68 patients found that supplementation with 20,000 IU vitamin D(3) weekly did not result in beneficial effects on clinical MS-related outcomes [28].

The present study is akin to prior trials in duration and sample size. We found no trend of clinical improvement regarding disease activity in PwMS after vitamin D supplementation; however MRI outcomes were not included in this study. Since patients participating in our study were all but one under long-term IFN-β treatment, disease activity as assessed by relapse rate, relapse numbers and EDSS change was as expected relatively low. The study was therefore underpowered to assess the effect of vitamin D on MS activity, and larger scale trials and meta-analysis of available data, including MRI parameters, are needed to clarify the benefit of vitamin D on MS disease activity and/or its symptoms.

IL-17 is a pro-inflammatory cytokine produced by Th-17 cells, and has been advocated to play a role in MS pathogenesis [29]. IL-17 has been found to be highly expressed in MS lesions, and Th17 cells are abundant in active MS lesions [30]. Vitamin D has been shown to decrease Th-17 cell activity in animal models of MS [31,32], and to down regulate IL-17 production in Th17 cells isolated from MS patients [33]. IFN-β was also shown to inhibit Th17 cell development, and this effect is considered to be one of the mechanisms of actions of IFN-β in the treatment of MS [34].

Since both vitamin D and IFN-β have been shown to down regulate IL-17 production, we premised that vitamin D supplementation would decrease IL-17 serum levels in our IFN-β treated patients. Unexpectedly, a significant increase in serum IL-17 was found in the low dose group of this study, while non-homogenous trends in IL-17 levels were noted in the high dose group. Due to ethical considerations, this study did not include a pure placebo group, without any kind of vitamin D supplementation. Therefore, it is impossible to determine from this data, whether the demonstrated increased IL17 was due to the low dose supplementation, or the consequence of some other unrecognized confounder. Since accumulating data point towards both vitamin D and IFN-β as inhibitors of Th17, it’s more probable that the increased IL-17 in the low dose group in this study was not due to vitamin D. Notably, baseline measurements were carried out at winter while 3 months tests were done during spring. Seasonal variation in immune cell subsets and cytokines has been previously described, and could have contributed to the variation in serum cytokines in this study [35,36].

The trend towards decreased IL-17 secretion in an increasing proportion of patients on higher pharmacological doses of vitamin D is in agreement with earlier studies which demonstrated that high dose vitamin D decreases Th-17 cell activity in animal models of MS [31,32] as well as in PwMS [33]. Beneficial effects of vitamin D increase may be undetectable in IFN-β treated patients, because of the strong immunomodulatory effect of this cytokine [9], however our results indicate that the add on of large doses of vit D could be beneficial. The question remains whether further increasing vitamin D dose, above 4,370 IU per day, would entail significant IL17 down-regulation in a greater proportion of MS patients.

Furthermore, IL-17 data must be interpreted with caution as serum IL-17 is not an established biomarker of MS disease activity. IL17 serum levels before treatment with IFN−β, as well as 3 months after treatment, could not be correlated to disease activity parameters in a cohort of 118 MS patients [37], and IL‑17 levels showed a trend towards being higher in MS patients with inactive disease compared to those with active disease in another patient cohort of 169 patients [38].

No change in serum levels of other cytokines was noted in this work, as well as in a previous study [39], assessing the impact of vit D supplementation.

Notably, PTH levels decreased significantly in the high dose vitamin D treated group. This change in PTH level was found also in healthy people after vitamin D supplementation [40]. The role of PTH as a potential immune-modulator has been debated. Both B and T lymphocytes express PTH receptors [41].While some studies imply that PTH may be anti-inflammatory [41] other studies found that PTH is in fact increased during MS relapse [42]; still others found no correlation at all between PTH levels and immune cells function [43]. Nevertheless, PTH is a well known stimulator of bone resorption. Its decrement in our patients who received the high dose of vitamin D may imply an advantage, though yet to be confirmed, in terms of bone health and prevention of osteoporosis, which is particularly common and debilitating in PwMS [44].

## Conclusions

The present randomized, double-blind, placebo-controlled trial, though modest in its sample size, did not detect beneficial effects of vitamin D supplementation on IFN-β -related FLS in PwMS, but did provide support to its immunomodulatory properties. Vitamin D appears to influence IL-17 secretion in IFN-β -treated patients in a dose dependent manner. While serum IL-17 was significantly increased after low dose vitamin D treatment, heterogeneous responses were noted after high dose vitamin D.

The findings are in-line with a series of clinical trials of vitamin D supplementation for PwMS, which generally did not show added benefit in terms of clinical efficacy, but did show clues for improvement in markers of inflammation and related MRI findings, beyond the reported effects on disease prevention [45,46]. Further large scale trials and meta-analyses of available data are needed to elucidate the role of vitamin D for immunocompetence and as part of the treatment armature of immune-mediated diseases as MS.

## Abbreviations

FAMS: ‘Functional assessment of MS’ questionnaire; FLS: Flu-like symptoms; HRQoL: Health related quality of life; IFN-β: Interferon beta; PwMS: Patients with multiple sclerosis; QoL: Quality of life; 25-OH-D: 25-Hydroxy-D.

## Competing interests

The authors declare that they have no competing interests.

## Authors’ contributions

DG participated in the design of the study, reviewed the literature, coordinated the study, performed clinical evaluations of the patients, participated in statistical analysis and wrote the manuscript. BH participated in the coordination of the study and in clinical evaluations of the patients. LGM participated in the design and coordination of the study, carried out the immunoassays and revised the manuscript. ESR participated in the design of the study, carried out the immunoassays and revised the manuscript. RO participated in the design of the study and carried out vitamin D and PTH assays. IL participated in the design of the study and performed statistical analysis. SD participated in clinical evaluations of the patients. MB participated in the design of the study. SIS participated in the design of the study and revised the manuscript. AM conceived the study, participated in its design, participated in clinical evaluations of the patients and revised the manuscript. All authors read and approved the final manuscript.

## Pre-publication history

The pre-publication history for this paper can be accessed here:

http://www.biomedcentral.com/1471-2377/13/60/prepub
